# Treatment of Cutaneous Injuries of Neonates Induced by Drug Extravasation with Hyaluronidase and Hirudoid

**Published:** 2014-07-03

**Authors:** Ya-Min Yan, Qiao-Ling Fan, Ai-Qiu Li, Jia-Ling Chen, Fei-Fei Dong, Mei Gong

**Affiliations:** 1 Neonatal Department; 2 Nursing Department; 3 Pediatric Intensive Care Unit; 4 Cardiac Intensive Care Unit, Shanghai Children’s Hospital, Shanghai Jiao Tong University, Shanghai, China

**Keywords:** Treatment, Drug Extravasation, Hyaluronidase, Hirudoid, Neonate

## Abstract

***Objective:*** To analyze the effects of hyaluronidase and hirudoid treatment on drug extravasation in neonates.

***Methods:*** The medical records of 13 neonates with drug extravasation treated with hyaluronidase and hirudoid between August 1^st^, 2010 and May 1^st^, 2012 were analyzed retrospectively. The treatment procedure for drug extravasation adhered to the protocol in neonatal department. The information including age, sex, weight, diagnosis, size of affected area, site of extravasation and treatment was collected.

***Findings*** ***:*** The extravasation injuries alleviated and the symptoms improved after treatment, no adverse drug effects were reported with use of hyaluronidase and hirudoid.

***Conclusion:*** The treatment appeared to be beneficial in the management of extravasations of various medications in neonates and may be useful in reducing the severity of cutaneous toxicosis. However, further studies with large samples are still needed to assess the effectiveness and safety of hyaluronidase and hirudoid.

## Introduction

Extravasation is the non-intentional leakage of infused fluid into surrounding soft tissue from the veins^[^^1^^]^ which may cause tissue damage, produce progressive necrosis of the skin and subcutaneous tissue^[^^2^^]^ and ultimately result in the malfunction or even amputation of the affected extremity^[^^3^^,^^4^^]^. The published incidence rates of drug extravasation range from 0.01%^[^^5^^]^ to 6.5%^[^^6^^]^. And the actual incidence rate is probably higher because of inconsistent documentation and report^[^^7^^]^. The children who receive intravenous (IV) administration therapy are more susceptible to extravasation injury than adults^[^^8^^]^. Neonates have additional multiple factors that may increase the risk of injury, such as poor venous integrity^[^^9^^-^^15^^]^, long duration of therapy, decreased peripheral circulation^[^^11^^]^, more flexible subcutaneous tissue^[^^12^^,^^13^^]^ and high frequency of receiving IV therapy through peripheral veins^[^^14^^]^. Besides, this population may not be able to localize and complain of pain in time. 

 The extravasation can cause further complications in the course of the disease and may lengthen the patient's hospital stay^[^^15^^]^ associated with significant morbidity in neonates^[^^16^^,^^17^^]^. Although nurses do their best to prevent extravasation, it still may occur as long as the IV therapy is employed.

 Hyaluronidase is an important enzyme agent used to increase the absorption and dispersion of other extravasation drugs^[^^18^^-^^20^^]^. It has also been used as an adjunct for the treatment of extravasation of chemotherapeutic agents^[^^21^^]^. According to the report, treatment with hyaluronidase is efﬁcacious for extravasation of various agents in pediatric population, including neonates. However, evidence-based guidelines for the treatment are still lacking^[^^22^^]^. The FDA approved Hylenex recombinant (hyaluronidase human injection) as an adjuvant agent to increase the absorption and dispersion of other injected drugs in 2005, and literature has shown beneficial outcomes. But the optimal management of intravenous extravasations remains contro-versial^[^^7^^]^. In a survey conducted in 551 neonatal intensive care units, only 57% extravasation had a procedure of injecting hyaluronidase, and 33% did not use it at all^[^^23^^]^. The exact dosage of hyaluronidase is still under debate (ranging from 15 units to 1,500 units with saline ﬂushout). A dose of 15 units is effective for treating extravasations in pediatric patients. However, a dose of 150 units are recommended for chemotherapy extravasations or extravasation with grade 3/4 level^[^^24^^]^. Hirudoid cream with major component being Mucopolysaccharide Polysulfate, is a local anti-coagulant agent. When applied to the skin, it can relieve pain, swelling, hematoma and inflammation. However, whether it can be used on children under the age of 5 years is still controversial. This paper demonstrates the treatment with hyaluronidase and hirudoid for drug extravasation in 13 neonates.

## Subjects and Methods

Hyaluronidase was purchased from Shanghai NO.1 Biochemical & Pharmaceutical Co., Ltd (Shanghai, China). Mucopolysaccharide polysulfate cream (hirudoid) was purchased from Mobilat Produktions GmbH (Bavaria, Germany).

**Fig. 1:**
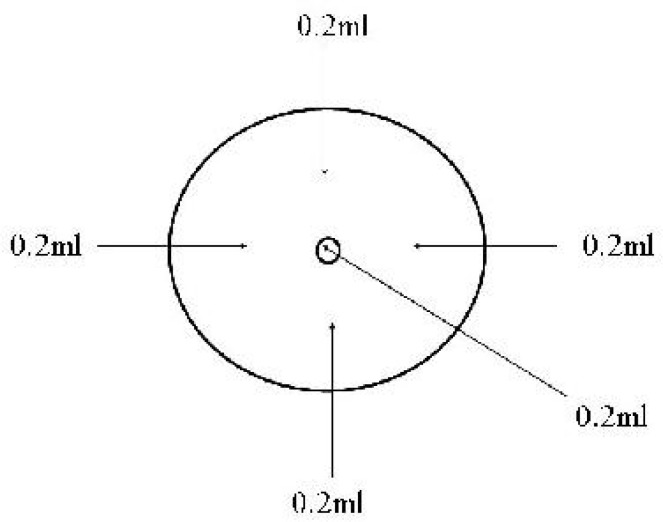
Amount of hyaluronidase and site of injection.

 This study was done in the neonatal department, Shanghai Children' Hospital between August 2010 and May 2012. The data of the patients who had experienced drug extravasation and were treated with hyaluronidase and hirudoid were collected. Once extravasation noticed, the infusion was stopped immediately, followed by a prompt evaluation by the vein management team, including degree of extravasation and cutaneous injuries, treatment methods and frequency of injecting hyaluronidase, etc. The procedure of injecting hyaluronidase was as follows: a 25-gauge needle was used and a total of 1-ml solution (150 U/ml) of hyaluronidase was divided into 5 0.2-ml injections: one in the center while 4 along the edge of the extravasation sites (Fig. 1). The needle was changed with each injection. If the cases needed another injection several hours after the first injection (contained five 0.2cc injections), to prevent its deterioration the “frequency of injecting” was evaluated. For example, in 2 cases with signs of necrosis, injecting hyaluronidase was repeated every 8 hours, its “frequency of injecting” was one injection per 8h, and the total dose of injection was 1 ml (containing five 0.2 cc injections). And in one day, it was injected 3 times, with a total dose of 3ml. All the procedures mentioned above were in accordance with the standards of the Committee on Human Experimentation of Shanghai Children' Hospital.

## Findings

In this retrospective analysis, there were a total of 13 cases, of which 8 were males and 5 females (Table 1). They were aged from 5 to 150 days, with a mean age of 25.54 days. The mean weight was 2.85kg. There was obvious swelling in the area of extravasation among all the patients: 5 (38.46%) had erythema, 3 (23.08%) had blister and 1 (7.69%) had tissue necrosis. 

 In 2 cases, a total of 1 ml hyaluronidase solution was injected according to the procedure, and hirudoid was massaged around the affected area every 6 hours for 2 days. In 10 cases, the procedure of injecting hyaluronidase was repeated every 12 hours for 3 times, and hirudoid was massaged every 6 hours for 4 days. In 1 case with signs of necrosis, injecting hyaluronidase was repeated every 8 hours for 3 days, hirudoid was massaged every 6 hours for 4 days and repetitive daily saline dressing was employed for 2 days.

 The efficiency was assessed according to the patients’ condition. In 12 cases, the hyaluronidase was given within 7 hours, with a median time of 6.39±1.35 hours. After the treatment, the symptoms improved, and no complications were noted at the follow-up within 48 hours and 3 months. Three cases were lost to follow-up. Negligible loss of functional movements of the fingers, hands, arms, feet, or legs was noticed. In one case, the medication involved was calcium chloride, and the hyaluronidase was given after 14 hours. Skin scar developed 2 days after the treatment, and calcinosis developed 3 weeks after he was discharged from the hospital. 

## Discussion

Intravenous administration of medications was always accompanied by the risk of extravasation. Drug extravasation is considered a medical emergency which can lead to serious outcomes if not treated appropriately^[^^8^^]^. It has been reported that the neonates are at a high risk of extravasation and subsequent injuries^[^^11^^]^, particularly in the first 2 weeks after birth, and about 15% of extravasations developed to skin necrosis^[^^25^^]^. The severity of extravasation should be assessed, and an emergency approach adopted before any signs of skin necrosis appear.

 Different therapeutic options have been evaluated in the management of extravasation. With local administration of hyaluronidase within the first 24 hours the best results will be obtained^[^^26^^,^^27^^]^. Many animal experiments and clinical reports have documented its effectiveness in reducing severity of skin damage and shorting of the healing time after extravasation^[^^21^^,^^28^^-^^31^^]^. In the present case, we used hyaluronidase because of its effects on enhancing distribution and absorption by breaking down hyaluronic acid. However, infiltration with hyaluronidase is an invasive procedure and British National Formulary has advised caution in the use of hyaluronidase in infants and to control the speed and total volume of injection. According to the report, the dose of hyaluronidase used in neonates ranges from 15U/ml^[^^32^^]^ to 1500U in combination with saline ﬂushing techniques^[^^14^^]^. The total dose is always divided and injected in 4 sites along the leading edge of the extravasation^[^^33^^]^. 

**Table 1:** Patients demography of 13 neonates with cutaneous injuries after drug extravasation

**Characteristics**		**No. (%) of Patients **
**Age**		25.54 days (5-150 days)
**Age group**	**preterm infant** **term infant**	5 (38.46) 8(61.54)
**Weight**		2.85 kg (1.3-3.95 kg)
**Male/female**		8 (61.54) / 5(38.46)
**Locations**	**general neonatal ward** **neonatal intensive care unit**	5 (38.46) 8 (61.54)
**Admission diagnosis (n=13)**	**Prematurity** **pneumonia** **gastrointestinal disorders** **severe malnutrition** **neonatal jaundice**	5 (38.46) 4 (30.77) 2 (15.39) 1 (7.69) 1 (7.69)

In our hospital, infants, smaller extravasations, grade1/2 extravasations are treated with 15 units, and chemotherapy, larger extravasations and grade 3/4 extravasations are treated with 150 units. However, this may be revised with the continual evaluation of the patients’ condition, extravasation drugs, extravasation sites, etc. In this study, there was apparent skin damage and necrosis in the extravasation site, so all the neonates received an injection of 150U/ml once and no adverse reactions were reported. 

 Risks for extravasation are multifactorial. It has been reported that medications with high osmolarity, low pH, and high molecular weight are more likely to cause extravasation injuries^[^^4^^]^. In this study, the most commonly used medication causing extravasation was total parenteral nutrition (TPN) solution, accounting for 69.23% (Table 2). This may be related to the fact that most neonates, especially in neonatal intensive care unit, always receive intravenous therapy of parenteral nutrition. The use of central venous catheter (CVC) for TPN has been a standard procedure since its introduction^[^^34^^]^, but the CVC or peripherally-inserted central catheter (PICC) cannot be used in all the neonates because of venous conditions, costs and safety. According to the advice, the medications with an osmolarity lower than 900mmol/L can be delivered through peripheral vein^[^^35^^]^. We took the TPN as high-risk medication, because the solution’s osmolarity in these cases was between 594 mmol/L and 844 mmol/L, higher than the normal level (280-310 mmol/L). 

 A site, which is convenient to observe and fastened securely must be chosen before IV therapy. However, there is not always appropriate site for cannulation. As the most common location for IV therapy, the dorsum of hands is always associated with extravasation. In this study, the dorsum of hands was involved in 4 (30.77%) cases, the leg and forearm in 3(23.08%) cases, and the wrist, armpit and scalp in 1 (7.69%) case. The size of extravasation area ranged from 1 cm^2^ to 60 cm^2^ with an average size of 19.23 cm^2 ^(Table 3).

 In this study, the most common admission diagnosis was prematurity, accounting for 38.46%. The reasons may lie in that the preterm infants have immature skin which is easily damaged, require longer duration of intravenous therapy and in whom venous puncture is not an easy task. The population is more susceptible to extravasation injuries^[^^36^^]^. Cosmetically or functionally serious scars caused by extravasations have been noted in 4% of premature infants^[^^7^^]^. Eight cases (61.54%) occurred in the neonatal intensive care unit in this study. However, there are few reports concerning the management of drug extravasation in the intensive care unit, so the exact managerial approaches are not well known. Nurses have important responsibilities in prevention and management of complications caused by IV usage, and taking optimal measures in cases of extravasation development^[^^37^^]^. So measures should be adopted to enrich knowledge of extravasation among nurses.

**Fig. 2:**
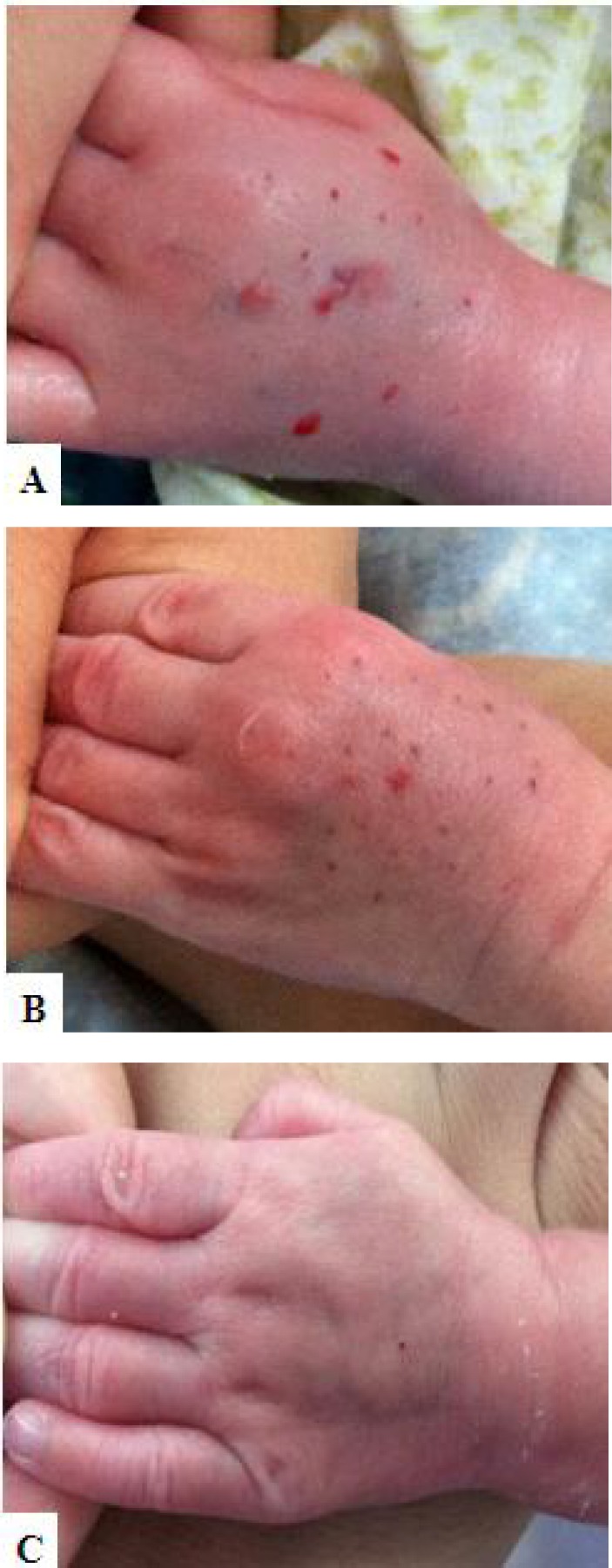
Use of hyaluronidase together with hirudoid decreased the severity of extravasation injuries. **(A)** Skin swelling and erythema a few hours after total parental nutrition fluid extravasation in a newborn aged 11 days. **(B)** Appearance 2 days after early treatment (<8 h). **(C)** Skin swelling and erythema completely disappeared in the 8th day after treatment, necrosis has been avoided.

 Treatment with hyaluronidase together with hirudoid can prevent skin necrosis in the neonates (case 11). There was apparent swelling and erythema in the dorsum of hands (Fig. 2A). The treatment procedure was efficient in preventing further damage to the skin. Skin swelling and erythema were alleviated (Fig. 2B) and completely disappeared on the 8^th^ day after treatment (Fig. 2C). It had no adverse impact on the activity and physiological functions in the follow-up of 1 month in this case. 

 Hyaluronidase and hirudoid must be used as early as extravasation was noticed. 

**Table 2:** Medications involved in extravasation in 13 neonates

**Medications**	**No.(%) of extravasation**
**Total parental nutrition**	9 (64.30)
**Calcium chloride**	1 (7.14)
**10% dextrose in water**	1 (7.14)
**IV immunoglobulin**	1 (7.14)
**PAMBA+etamsylate**	1 (7.14)

PAMBA: para-aminomethylbenzoic acid

**Table 3:** Cases with site and size of the involved area in extravasation

**No.**	**Age (days)**	**Sex**	**Site**	**Size (cm** ^2^ **)**
1	7	Male	wrist	1×2.5
2	24	Male	dorsum of hand	2×1.5
3	25	Female	forearm	1×3
4	8	Male	scalp	3×3
5	18	Male	dorsum of hand	1.5×3
6	21	Male	leg	1×1
7	150	Male	forearm	4×3
8	14	Female	leg	4×4
9	5	Male	leg	7×7
10	17	Female	armpit	7×7
11	11	Female	dorsum of hand	4×4
12	20	Female	forearm	12×5
13	12	Male	dorsum of hand	5×5

According to the experience of vein management staffs in three Shanghai pediatric hospitals, the golden time for injecting hyaluronidase is 8 hours, and 3 hours for high risk medications, such as dopamine and epinephrine. But if exceed the golden time and the extravasation has done damage to the patient or developed to grade 3 or grade 4, hyaluronidase is still injected to prevent further damage, and has been effective. 

**Fig. 3:**
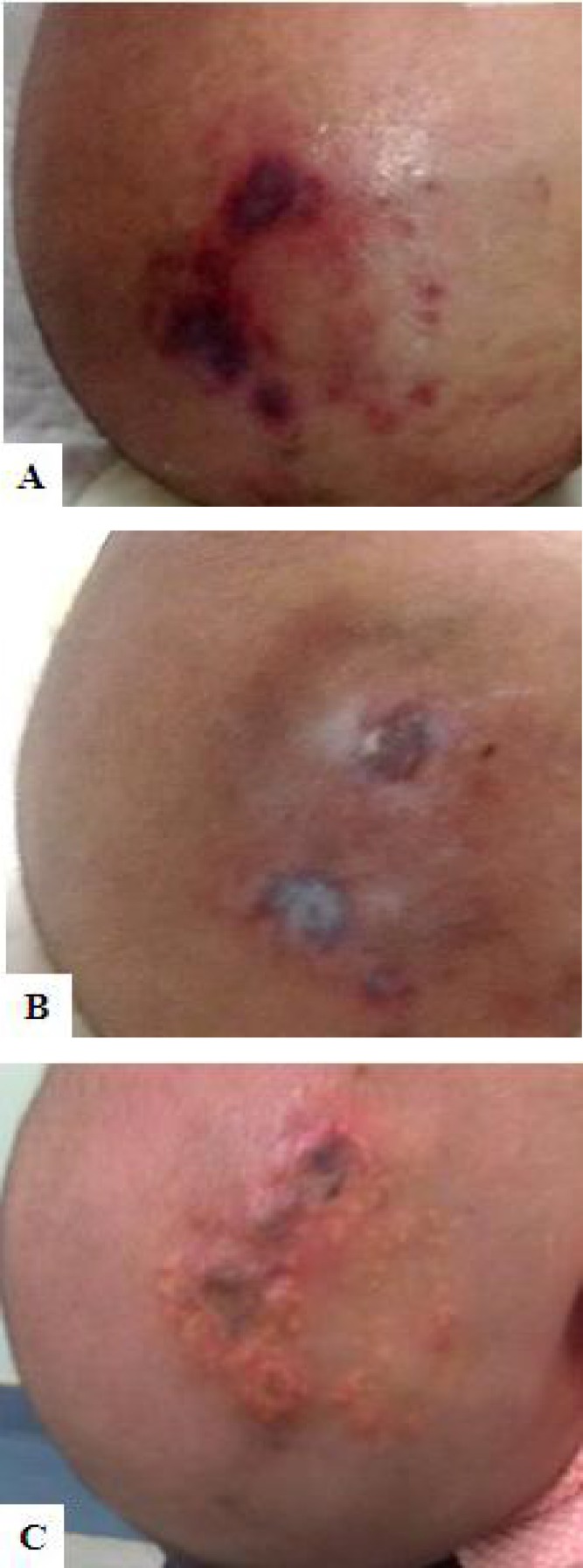
Time of treatment initiation influences the effect. **(A)** Appearance of extravasation of calcium chloride on the scalp, treatment was given more than 14h after the extravasation. **(B)** Skin scar was developed 3 days after the event happened. **(C)** Calcinosis was developed 3 weeks after discharge from hospital.

However, if the extravasation has happened longer than 72 hours, injecting hyaluronidase has little effect. In 12 cases of this trial, the median time of treatment was 6.39±1.35 hours, within 7 hours after the extravasation happened, and good results have been obtained. In one case (case 4), the scalp was severely damaged by extravasation of calcium chloride (Fig. 3A), hyaluronidase and hirudoid were given 14 hours after the extravasation. Apparent skin scar was developed 3 days after the treatment (Fig. 3B), and the patient was discharged from the hospital with minor scar 6 days later. Health guidance for the scar and usage of hirudoid was delivered to the family. 

Unfortunately, the patient was readmitted to the hospital because of calcinosis (Fig. 3C) 3 weeks later, and corresponding interventions were adopted immediately. This indicated that the nurses should pay more attention and check the patients more times when infusion is continuing, especially to the patients who receive high risk medications.

## Conclusion

This article demonstrates an encouraging result of drug extravasation managed by hyaluronidase and hirudoid within 8 hours after it occurred in neonates. The use of hyaluronidase together with hirudoid should be considered for the management of various drug extravasations.
